# Preparation of Esterified Starches with Different Amylose Content and Their Blending with Polybutylene Succinate

**DOI:** 10.3390/ijms25126301

**Published:** 2024-06-07

**Authors:** Shuning Liu, Shi Tang, Yuanhao Lu, Tingting Su, Zhanyong Wang

**Affiliations:** 1 School of Petrochemical Engineering, Liaoning Petrochemical University, Fushun 113001, Chinatangshitangshi2024@outlook.com (S.T.); luyuanhao001@outlook.com (Y.L.); 2 College of Bioscience and Biotechnology, Shenyang Agricultural University, Shenyang 110866, China

**Keywords:** esterified starch, polybutylene succinate, degradation, melt blending

## Abstract

Three types of starch with different amylose content were esterified and blended with polybutylene succinate (PBS) to obtain esterified manioc starch/PBS (EMS/PBS), esterified corn starch/PBS (ECS/PBS), and esterified waxy corn starch/PBS (EWS/PBS) composites. The EMS/PBS and ECS/PBS composites with high amylose content displayed typical V-type crystal structures. The original crystals of EWS, which had low amylose content, were disrupted during the esterification process. EWS exhibited the strongest interaction with PBS and the most favorable interface compatibility. The pyrolysis temperature was in order of EMS/PBS < ECS/PBS < EWS/PBS. The elongation at break of the three blends was higher than that of pure PBS. The esterification and plasticization of the EWS/PBS composite were the most comprehensive. The EWS/PBS composite showed the lowest storage modulus (G’) and complex viscosity (η*). The interfacial bonding force of the composite materials increased with more amylopectin, decreasing intermolecular forces and destroying crystal structures, which decreased G’ and η* and increased toughness. The EWS/PBS composite, with the least amylose content, had the best hydrophobicity and degradation performance.

## 1. Introduction

The environmental problems caused by ‘white pollution’ are becoming increasingly serious, and the development of biodegradable materials is one of the practical ways to solve this problem [1]. Polybutylene succinate (PBS) is the most promising fully biodegradable plastic with a wide range of applications [2]. However, the high crystallinity, low processing temperature, and high production cost of PBS limit its application. Biodegradable polymers can be blended with naturally degradable materials, such as starch, to obtain composites with degradable properties and reduce the costs [3]. The blending of degradable plastics with starch has received widespread attention. Lai et al. compared the different properties of PBS blending with untreated starch and gelatinized starch, indicating the gelatinized starch could improve the compatibility, resulting in a 2-fold increase in tensile strength and a 1.5-fold increase in elongation at break compared to the untreated starch/PBS composites [4]. Li et al. found that waxy starch/PBS has greater advantages in processability, mechanics, and hydrophobicity compared with corn starch/PBS blends [5]. A thermoplastic starch reacted with sodium metabisulfite and blended with poly(butylene adipate-co-terephthalate), which improved the transparency and hydrophobicity, as well as antimicrobial properties [6]. Phattarateera et al. investigated the effect of different contents of pregelatinized starch and hydrolyzed starch on the properties of polyvinyl alcohol (PVA) films, and the results showed that the mechanical properties of the two blends were increased [7]. Srihanam et al. used citric acid as a compatibilizer to improve the compatibility and mechanical properties of poly(L-lactide)-*b*-poly(ethylene glycol)-*b*-poly(L-lactide)/starch blends, and the results showed that the thermal stability, compatibility, and mechanical properties of the blends were improved after modification by citric acid [8]. Compared with the native starches, the interfacial interaction and hydrophobicity of the composites with esterified starches had been improved [9]. Esterified native polymer is promising for packaging materials, the food industry, and the production and manufacture of degradable materials [10,11,12].

Manioc starch (MS), corn starch (CS), and waxy corn starch (WS) are the most common and inexpensive starches. Using the three starches to modify PBS can reduce production costs and could be widely used in packaging materials, etc. By adding esterified starch to PBS, it is possible to improve the mechanical properties, reduce the crystalline properties, and enhance the degradation properties. Furthermore, the properties of esterified starch/PBS composites are affected by the content of straight-chain starch. Although many scholars have investigated esterified starch/PBS composites, few works studied the differences in the properties of PBS blended with different amylose contents of esterified starch. The present work aims to investigate the performance differences of esterified starch with different linear chain contents after blending with PBS for modification.

## 2. Results and Discussion

### 2.1. Evaluation of Starch Esterification

**Figure 1:**
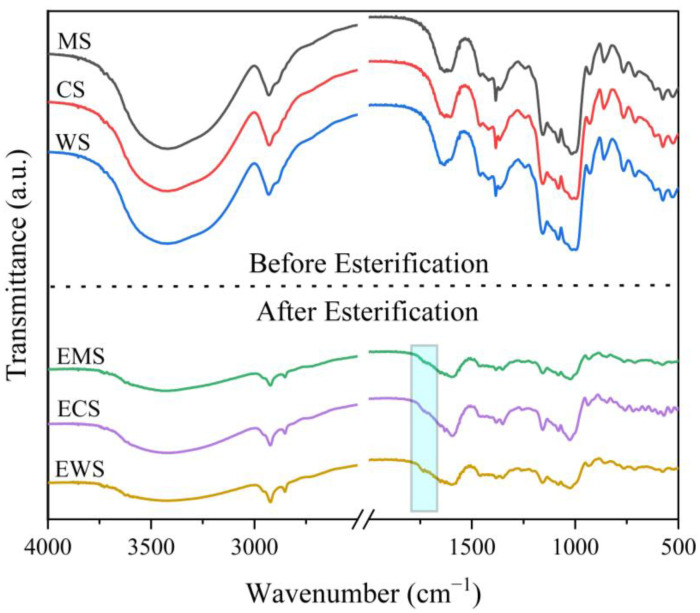
FTIR of the native starches and esterified starches.

Fourier transform infrared spectroscopy (FTIR) was used to characterize natural and esterified starches to determine whether the esterification reaction occurred completely or not, and the results are shown in Figure 1. Compared with the natural starch, the infrared spectra of esterified starch contained a C=O absorption peak at 1735 cm^−1^. The carbonyl formation can be determined based on the location of the absorption peak, which indicates the occurrence of esterification [13]. The intensity of the characteristic peak of -OH reduced, which indicated the strength of the hydrogen bond was weakened during esterification [14]. The three starches were successfully esterified, and MS had the same amount of hydrogen bonds compared with the other two esterified starches. Therefore, the plasticizing effect and processability of the EMS may be relatively poor.

### 2.2. Crystal Structure Analysis

**Figure 2:**
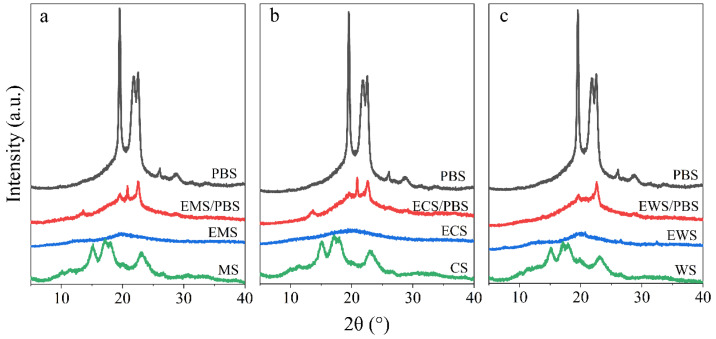
X-ray diffraction (XRD) of PBS, native starches, esterified starches, and esterified starch/PBS blends. ((**a**): the system of MS; (**b**): the system of CS; (**c**): the system of WS).

The hydroxyl groups in starch molecules are replaced by ester groups during the esterification reaction, causing the crystal structure of the starch to be destroyed. As shown in Figure 2, the position of the diffraction peaks of esterified starch changes, and the corresponding intensity also decreases. According to the curves, the lowest crystallinity of the natural starch and esterified starch is 25% and 5%, respectively. It can be concluded that the esterification destroys the crystal structure of the native starch. The amount of hydroxyl groups substituted in amylose varies depending on the content of linearity. Generally, the molecular chains of the linear starch are more prone to crystallization due to their tight arrangement [15]. Esterification has a certain destructive effect on the crystal structure, reducing the crystallinity of starch. The crystallinity of starch is related to the content of amylose. The crystalline structure of esterified starch/PBS composite is largely influenced by that of esterified starch. The blended system of three esterified starches exhibited characteristic diffraction peaks at 19.5° and 22.5°. The thermoplastic starch using glycerol as a plasticizer has two distinct crystal forms: one is the residual crystalline structure generated by incomplete melting of starch, and the other is the V-type crystalline structure formed by amylose starch [16,17]. The diffractogram of EWS did not show peaks at 13.5° and 21°. The original crystals of WS were destroyed during the esterification process. EMS/PBS and ECS/PBS composites exhibited classical V-type crystal diffraction characteristic peaks at 13.5° and 21°. The V-type diffraction peaks were formed by single helix linear starch crystals during extrusion processing [18,19]. Amylose starch affects the complete plasticization of starch and the formation of V-type crystalline; therefore, the type of starch affects the crystal structure of thermoplastic starch.

The EWS/PBS composite had a smaller peak area than the EMS/PBS and ECS/PBS composites, indicating that EWS had the strongest interdependence with PBS and the best interface compatibility. On the one hand, the hydroxyl group (hydrophilic) on the starch molecular chain of WS was replaced by the ester group (hydrophobic) and enhanced the interfacial compatibility of the PBS after esterification. On the other hand, the crystal structure could be greatly damaged, with the highest thermoplastic properties and enhanced dispersion when blended with PBS. The crystallization region was destroyed during the esterification, resulting in a decrease in crystallinity, which improved its thermoplastic properties and subsequently significantly increased the dispersibility and dependence of the material.

### 2.3. Morphological Observations

**Figure 3:**
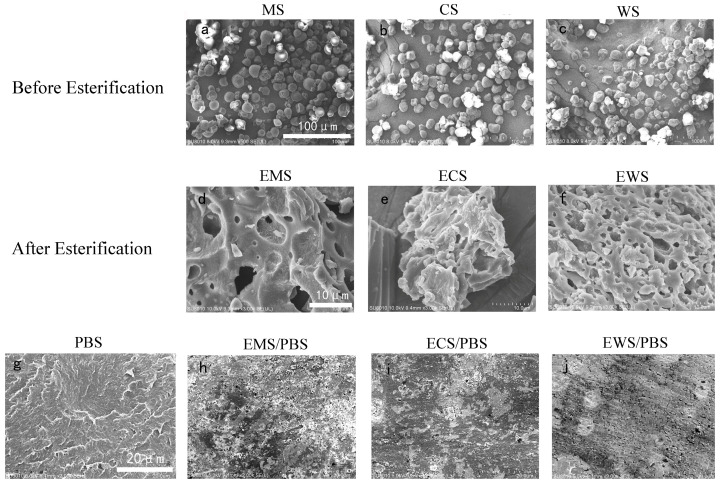
The fracture surface of the native starches, esterified starches, and esterified starch/PBS blends.

Figure 3 shows the scanning electron microscopy (SEM) images of the three kinds of esterified starch and PBS blends. The external form of natural starch is usually a solid, rounded structure with very smooth, well-defined corners. After esterification in the solution, starch undergoes the process of pasting. The central region around the nucleus of the starch is the weakest ordered region [20]. Its structure is destroyed; therefore, the densification deteriorates, fragmentation increases and the outline is blurred [21]. After the esterification of MS with the highest content of straight chains, the system was slightly fragmented, and the fragment agglomeration was more obvious after the esterification of WS with the highest content of branched chains. This may be due to the more branched chains disrupting the dense and homogeneous gel network structure of the starch itself. The addition of plasticizers can better improve the thermoplasticity of the material. When the starch particles were plasticized, the hydrophilic hydroxyl groups in the esterified starch were replaced by hydrophobic ester groups. This reduced intermolecular forces and improved thermoplastic properties. The ECS/PBS composite had no obvious particles, and a large amount of incompletely plasticized starch fragments existed in the blend (Figure 3i). The cross-section of the EWS/PBS composite did not present starch particles but a large number of cracks (Figure 3j). This is because the crystallization of the esterified starch is severely disrupted and the molecular chains are too loose.

### 2.4. Thermal Performance Analysis

**Figure 4:**
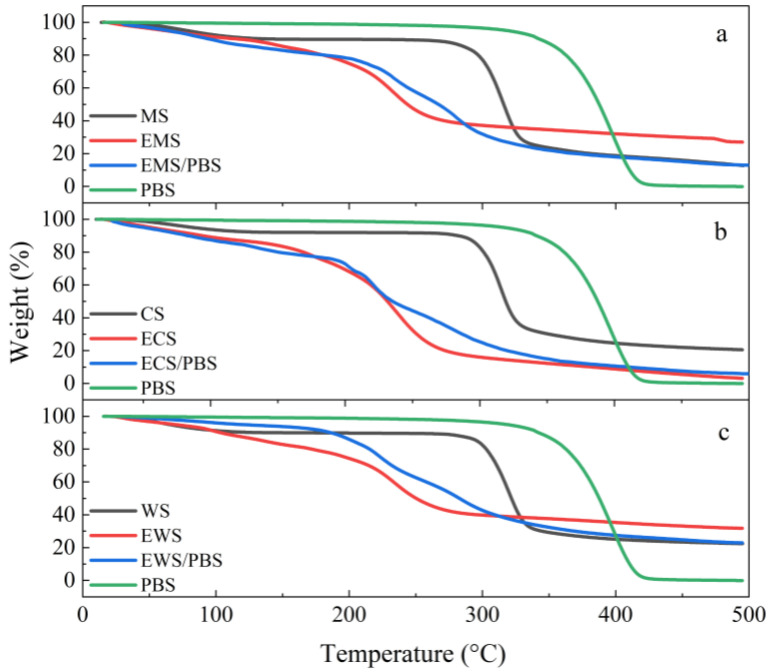
Thermogravimetric analysis (TGA) of the native starches, esterified starches, and esterified starch/PBS blends. ((**a**): the system of MS; (**b**): the system of CS; (**c**): the system of WS).

The thermal decomposition behavior of the blends of three types of esterified starch and PBS was similar (Figure 4). Firstly, water and glycerol evaporated during the initial stages. Then, weight loss was caused by the thermal decomposition of the esterified starch. Lastly, weight loss was caused by the thermal degradation of the PBS, which occurred above 400 °C [4,22]. The weight loss of the composites was 77.1% (EMS/PBS), 87.1% (ECS/PBS), and 94.1% (EWS/PBS) at 500 °C, respectively. The order of weight loss rate was EMS/PBS < ECS/PBS < EWS/PBS. The inherent chemical structure of the starch molecules affected the insulation performance of the insulation layer, resulting in different levels of weight loss [23]. At the same temperature, the residual weight of the blends was 6–22% of the original weight, which was caused by starch carbonization [24,25]. As the content of amylopectin increased, the weight loss of the composites was accelerated. One of the reasons was that after esterification, the interface compatibility with PBS was increased, and the dependence between the two materials was increased. The second was that the crystallinity of the composite also decreased, resulting in a decrease in the temperature corresponding to the maximum rate of thermal decomposition.

### 2.5. The Properties of Esterified Starch/PBS Blends

#### 2.5.1. Tensile Test

**Figure 5:**
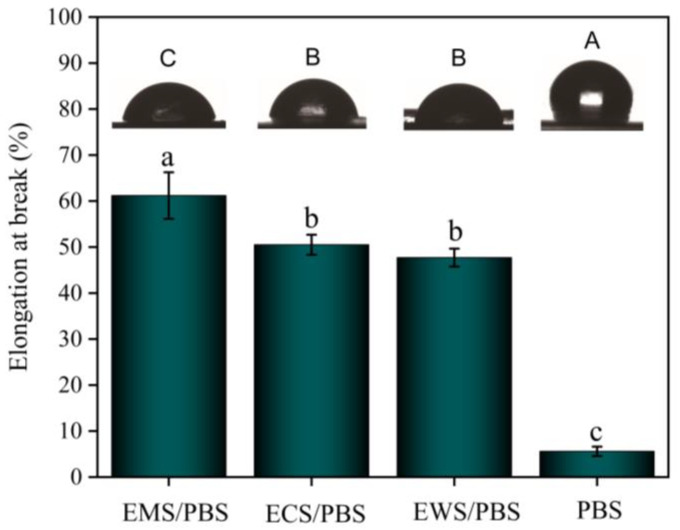
Break at elongation and WCA of the esterified starch/PBS blends. (The different lowercase letters indicate significant differences in elongation at break, while the different uppercase letters indicate significant differences in WCA).

After esterification, the crystallinity of the starch decreased, and the mechanical properties of the material changed. The ester groups contained in the esterified starch improved the interfacial compatibility and mechanical properties of the material. Figure 5 shows the elongation at break of the esterified starch composites. The fracture elongation of the three blends was higher than that of pure PBS. The increase in toughness was due to the decrease in the crystallinity of the starch. The optimal breaking elongation of the EMS/PBS composite with the highest amylose content was 61.2%, while that of the EWS/PBS composite with the lowest amylose content was 47.7%. The content of amylose was directly proportional to the breaking elongation of composite materials, which was consistent with previous research results [26,27]. Overall, the mechanical properties of all three composites were greatly improved but the difference was not significant.

#### 2.5.2. Water Contact Angle (WCA) Test

PBS is a hydrophobic material. However, thermoplastic esterified starch has a water absorption effect. The study focused on the influence of amylose content on the water absorption of the blend, which was mainly related to the degree of esterification and plasticization. As shown in Figure 5, the order of WCA was EMS/PBS (115.9°) < ECS/PBS (127.6°) < EWS/PBS (129.7°) < PBS (135.2°). After esterification, the hydroxyl groups on the molecular chain were replaced by ester groups, enhancing the interfacial adhesion and effectively preventing water molecules from penetrating the composite [28,29]. The esterification increased the water resistance of the material to a certain extent. As the content of amylose increased, the contact angle of the esterified starch/PBS composites gradually decreased. The gaps existing inside the material narrowed due to the improvement of compatibility, and the transfer of water molecules between hydroxyl groups was hindered due to the PBS chain segments. The entry of water molecules was inhibited due to the enhanced interfacial adhesion. The final experimental results indicated that the esterification and plasticization of the EWS/PBS composite were the most comprehensive.

#### 2.5.3. Dynamic Mechanical Analysis

**Figure 6:**
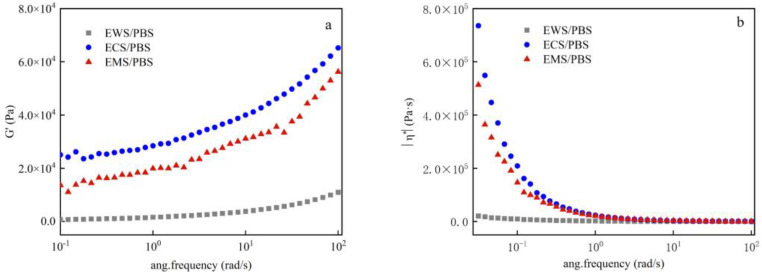
Energy storage modulus and viscosity of the esterified starch/PBS blends. ((**a**): G’; (**b**): η*).

Figure 6 shows the G’ and η* viscosity of the esterified starch/PBS blends. The ratio of amylose to amylopectin in the esterified starch had a certain impact on its properties. As shown in Figure 6, the G’ of the composites was proportional to the frequency. When the frequency of the composite was in a low-frequency state, the deformation exhibited viscous flow, and the energy loss of such changes was irreversible. When the frequency was at a high frequency, the viscous flow was too tight, and the change in elastic deformation was faster than its speed. Elastic deformation in this state was highly beneficial and mostly reversible. The higher the frequency, the greater the G’. At low frequencies, the trend of G’ became more and more obvious as the frequency changed, indicating that composite materials had obvious viscous characteristics. The η* of the composites was inversely proportional to the frequency. The main reason was that velocity gradients appeared between the liquid layers during the polymer flow process. If a large molecule passes through several liquid layers with different flow rates at the same time, the various parts of the same large molecule must advance at different speeds, which is not sustainable. Therefore, during flow, each long-chain molecule strived to fully enter the same flow-rate liquid layer, and the parallel distribution of different flow-rate liquid layers led to the orientation of the macromolecules in the flow direction, which led to a decrease in frequency during the flow process. When the ambient temperature of the PBS increased, the molecular motion speed and the volume increased, and the interaction between the two weakened, increasing the fluidity. After starch esterification modification, the shear thinning degree of the composite material decreased [30]. The G’ and η* of the ECS/PBS and the EMS/PBS composites gradually increased. This was because the esterification improved the interfacial bonding strength between the starch and the PBS and enhanced intermolecular interactions, and at this time, the degree of crystal structure damage was relatively small, resulting in an increase in G’, thereby increasing flow resistance. In addition, due to the higher content of branched starch in the ECS/PBS composite compared to the EMS/PBS composite, amylose molecules dissociated at 140 °C, causing them to entangle and form a strong network with branched starch, resulting in stronger “elastic” properties [26]. The EWS/PBS composite with the highest content of branched starch had the lowest G’ and η*. To summarize, the interfacial bonding force of the composite materials could increase with an increase in the number of amylopectins, leading to a decrease in intermolecular forces in starch molecules and the destruction of crystal structures, resulting in a decrease in G’ and η* and an increase in the toughness of the composite.

#### 2.5.4. Enzymatic Degradation

**Figure 7:**
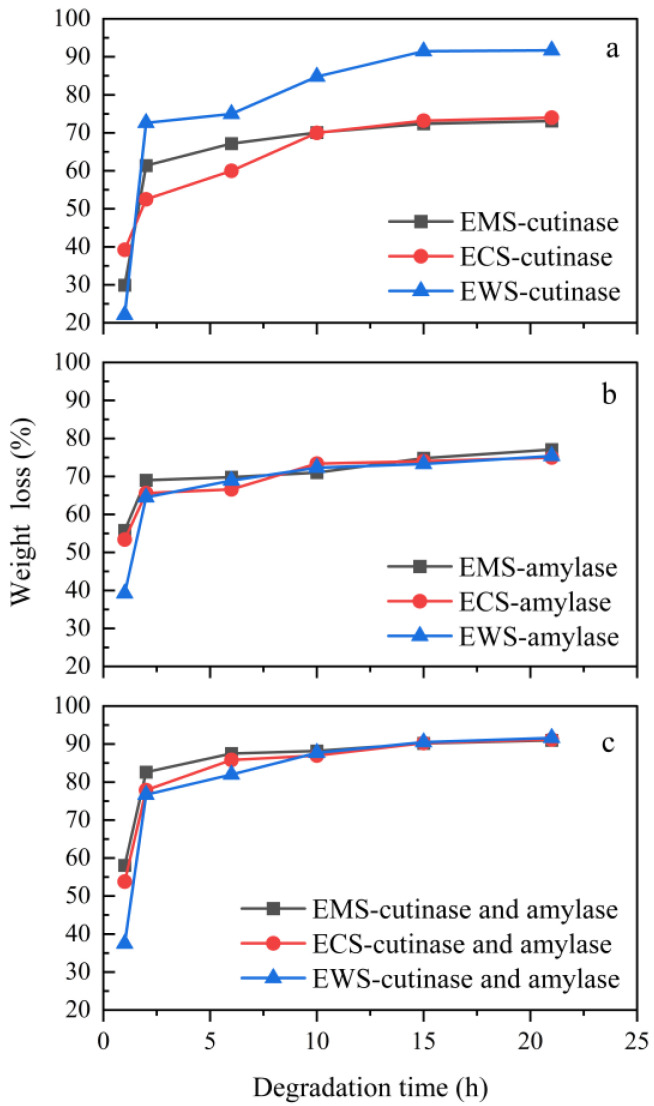
Weight loss of esterified starch/PBS blends degraded with cutinase and amylase. ((**a**): cutinase; (**b**): amylase; (**c**): cutinase and amylase).

Figure 7 illustrates the enzymatic degradation of the three blends with cutinase or amylase. When only cutinase was used as the catalyst, the weight loss of the composite increased rapidly within 3 h. In the first 3 h, the weight loss of the blend film was faster due to the cleavage of ester bonds in the PBS with cutinase, resulting in shorter polymer fragments. The EWS/PBS composite had a weight loss of up to 73% in the first 3 h, much higher than the 53% and 51% of the other two composites. The degradation slowed down in 3–21 h due to a copious of oligomers and small molecule substances in the buffer solution. The pH condition of the solution changed, and the suitable conditions for enzymatic degradation were disrupted, which was not conducive to the catalytic action of cutinase, resulting in a lower enzymatic degradation rate of the material [31]. The degradation rate of the EWS/PBS composite reached 91% after 21 h of degradation. The molecular structure of polyester gradually became loose and disorderly, which was beneficial for enzymes to attach to the ester bonds of polyester, thereby accelerating degradation. The effect of intermolecular forces gradually decreased, and the degradation performance gradually improved. This also confirms the above results and confirms the analysis of the best esterification effect of WS.

When only amylase was used as the catalyst, the weight loss of the composite increased rapidly within 3 h, and after the degradation time exceeded 6 h, the weight loss slowly increased. When the degradation time reached 21 h, the material quality loss reached about 75%. In general, the degradation of the composites in the enzyme buffer can include two forms: enzymatic hydrolysis and hydrolysis. Generally, when the two degradation methods work together, the weight loss in the composites increases [32]. The highest degradation efficiency of the EMS/PBS composite was 75%, which indicated that the esterification effect of MS was poor, and the compatibility with PBS was poor. Compared to the other two blends, there were more starch particles.

The degradation efficiency of the two enzymes, amylase and cutinase, was much higher than that of a single enzyme. When the two enzymes acted together, due to different targeting, the degradation did not affect each other. At 21 h of degradation, the degradation rate of the three composite materials was above 90%. Among them, the EWS/PBS composite was degraded best with both enzymes working together.

## 3. Materials and Methods

### 3.1. Preparation of Esterified Starch

PBS was obtained from Anqing He Xing Chemical Co., Ltd. (Anqing, China). MS was purchased from Guangxi Honghao Starch Development Co., Ltd. (Nanning, China). CS was purchased from Zhuhai Jindichao Food Co., Ltd. (Zhuhai, China). WS was purchased from Zhongsheng Food Ingredients Co., Ltd., Shantou, China. All three types of starch are food-grade. The amylose content of three types of starch is 65%, 55%, and 2%, respectively. *Fusarium solani* cutinase was prepared in our laboratory [17] and α-amylase from *Aspergillus oryzae* was purchased from Sigma, Garner, NC, USA.

### 3.2. Blending of Esterified Starch/PBS Composites

The formic acid (pH 2.5) and starch were mixed in a 1/0.17 ratio. The mixture was stirred thoroughly at 110 °C for 3 h. The product was then washed with anhydrous ethanol and dried at 45 °C to a constant weight. After crushing, grinding, and sieving, three types of esterified starch were obtained and named EMS, ECS, and EWS, respectively. Three esterified starch and glycerol were premixed in a 100/30 mass ratio for 20 min, respectively. Then, they were sealed and stored for 24 h to fully plasticize. The above mixtures were put into a torque rheometer (DHR-2, TA, New Castle, DE, USA) for extrusion mixing through an extrusion process, and then PBS was added in an 80/20 ratio. Each of the above processes was carried out at 140 °C for 15 min at 60 rpm.

The composite materials were preheated in a hot press at 150 °C for 2 min, then kept at a constant pressure of 50 kg/cm^2^ for 1 min. The blends were pressed at room temperature for 5 min to form a blended film with a thickness of 0.5 mm and 1.25 mm and dried to a constant weight.

### 3.3. Characterization of Esterified Starch and Esterified Starch/PBS Composites

#### 3.3.1. Fourier Transform Infrared Spectroscopy (FTIR)

FTIR spectra of esterified starches were recorded at ambient temperature with an infrared spectrometer (Agilent Cary 660, Agilent, Santa Clara, CA, USA). Each sample was scanned in the range of 4000–400 cm^−1^.

#### 3.3.2. Powder X-ray Diffraction (XRD) Analysis

XRD patterns were investigated using an X-ray diffractometry (D8 Advance, Bruker, Billerica, MA, USA) operating at 40 kV using Cu Kα radiation (λ = 0.1541 Å). X-ray diffractograms were recorded in the Bragg-angle (2θ) range from 5 to 40 degrees at a scanning speed of 5°/min.

#### 3.3.3. Scanning Electron Microscopy (SEM)

After promoting cooling with liquid nitrogen, the composite films were subjected to fracture treatment. The fracture surface of morphological observation was performed using a scanning electron microscope (SU8010, Hitachi, Tokyo, Japan) under an acceleration of 20 kV. All samples were covered with a thin layer of gold before testing.

#### 3.3.4. Thermogravimetric Analysis (TGA)

The thermal decomposition behavior of the composites was determined through TG analysis (TA, Q600, USA). The samples were heated from room temperature to 500 °C at 10 °C/min under a nitrogen atmosphere.

#### 3.3.5. Water Contact Angle (WCA)

A water contact angle measuring instrument (DSA 100, Krüss, Hamburg, Germany) was used for the determination of the contact angle. Static injection rate was 0.5 μL/s and injection volume was 3.0 μL. The sample was tested 5 times at room temperature (25 °C).

#### 3.3.6. Tensile Test

The test samples were cut into strips of 300 mm length, 4 mm width, and 1.25 mm thickness. The static tensile test was performed with a digital display electronic tensile testing machine (LDS-02, Chuanbai, Jinan, China) with a constant cross-head rate of 10 mm/min for all fabricated composite specimens. Tensile tests were performed at room temperature. Five samples were tested for each specimen and the average value was considered.

#### 3.3.7. Dynamic Mechanical Analysis

Rheological performance testing was performed using a rotary rheometer (DHR-2, TA, USA). It was used in a 25 mm parallel fixture with a sample thickness of 1 mm in oscillation mode. Strain scanning: 1 Hz, stress was 1 Pa, temperature scanning: frequency was 1 Hz, strain was 1%, dynamic frequency scanning: temperature was 140 °C, strain was 1%. The angular frequency was between 0.01 and 100 rad/s.

### 3.4. Enzymatic Degradation of Esterified Starch/PBS Composites

The composite films (30 mm × 10 mm × 0.1 mm) were incubated in 20 mM phosphate buffer (pH 7.4) with 0.2 mg/mL cutinase or α-amylase at 40 °C. After degradation for differing times, the films were cautiously gathered and rigorously washed with distilled water. Then the films were dried under vacuum until a constant weight. The weight loss was calculated as Eq. W_loss_ (%) = (W_0_ − W_D_) × 100%/W_0_ [31].

W_loss_ (%) is the weight loss of the composite films, W_0_ is the weight of the composite films before degradation, and W_D_ is the weight of the composite films after degradation.

### 3.5. Statistical Analysis

The statistical analysis was carried out using one-way analysis of variance (ANOVA) followed by Duncan’s multiple range test with SPSS 26 software. The values are presented as means ± SD and the results were considered as statistically significant at *p* < 0.05.

## 4. Conclusions

Three types of esterified starches with different straight-chain contents, EMS, ECS, and EWS, were blended with PBS. The influence of different starches on the properties of thermoplastic starch/PBS blends was analyzed. The EMS/PBS composite had the best toughness and mechanical properties. The EWS/PBS composite exhibited superior moisture resistance and degradation performance, making it a promising candidate for applications requiring enhanced durability and environmental degradability, including packaging materials, the food industry, and agricultural production. Such a result is due to the different proportions of amylose and amylopectin in the three types of starch. The content of amylopectin in the EWS/PBS composite was the highest, with the lowest intermolecular force, the smallest XRD diffraction peak area, the strongest interfacial compatibility, the highest G’, and η*, and the best degradation effect. To summarize, the characteristics of esterified starch are impacted by the amylose starch content, allowing for the manipulation of starch-based material properties by adjusting the amylose starch content.

## Data Availability

The datasets and materials used and/or analyzed during the current study are available from the corresponding author upon reasonable request.

## Abbreviations

PBS: polybutylene succinate; PVA: polyvinyl alcohol; MS: manioc starch; CS: corn starch; WS: waxy corn starch; EMS: esterified manioc starch; ECS: esterified corn starch; EWS: esterified manioc starch; FTIR: Fourier transform infrared spectroscopy; XRD: X-ray diffraction; SEM: scanning electron microscopy; TGA: thermogravimetric analysis; WCA: water contact angle.
